# Study on diffusion mechanism and failure behavior of epoxy coatings focusing on synergistic effect of temperature and water molecules

**DOI:** 10.1080/15685551.2021.1904581

**Published:** 2021-03-29

**Authors:** Chao Yang, Qing Han, Anquan Wang, Wei Han, Liang Sun, Laishun Yang

**Affiliations:** a Postdoctoral Scientific Research Workstation of Shengli Oilfield Company, SINOPEC, Dongying, China; b Technology Inspection Center of Shengli Oilfield, SINOPEC, Dongying, China; c College of Civil Engineering and Architecture, Shandong University of Science and Technology, Qingdao China)

**Keywords:** Temperature, epoxy coatings, two-stage diffusion, adsorption-desorption cycles, swelling coefficient

## Abstract

The research on the diffusion of water in coatings has been a hot topic in many fields, such as chemistry, coating structure and hydrogen bonding. In this paper, DGEBA epoxy coating was used as a sample to explore the diffusion process of 3 wt.% NaCl solution at different temperatures. Two-stage diffusion was established by immersion experiment and MD simulation. The synergistic effect of temperature and water on DGEBA properties was revealed by FTIR method and adsorption-desorption cyclic testing. The results showed that as the temperature increased, the saturation water content in coatings increased. At different temperatures, the diffusion process of water presented two-phase characteristics, and the influence of temperature on the diffusion process was mainly manifested in the cross-linking density at higher locations. Based on the variation law of swelling coefficient in per unit time (24 h), the conditions for water-coating interaction and formation of water clusters in this paper were proposed. The synergistic effect of water and temperature on DGEBA properties was reflected in two aspects: at lower temperature (20 °C), water would only change the physical structure of the coatings, while the water broke the DGEBA chains at higher temperature (60 °C).

## 1. Introduction

Organic coatings are widely used in metal corrosion protection, especially for buried metal pipeline, in which coatings not only make the pipe to reduce contact with the soil corrosive medium but also decrease the influence of external stress on pipeline [1].

In recent years, the coating failure caused by the diffusion of corrosive media has attracted extensive attention, which involves the relaxation process of coatings, electrochemical and stress analysis [2,3]. However, due to the complexity of coating structures and functional groups, the diffusion process of water, as well as relaxation process and the formation conditions of water clusters, has not been unified until now [4].

As the transport medium of oxygen and ions, the first problem to explain the diffusion process of water is to determine the diffusion path and the relaxation conditions of the coatings [3,5], but due to the complexity of the coating structures, it is difficult to analyze the above two problems in detail at the molecular level [2]. Relevant scholars have conducted a large number of studies on the diffusion process of water molecules in coatings focusing on the effect of temperature, coating crosslinking density, relaxation process and formation conditions of water clusters and established non-Fick diffusion processes, such as two-stage diffusion and S-type diffusion. However, the description of diffusion process only focuses on describing the macroscopic diffusion process, and few further expound the causes of the above-mentioned non-Fick diffusion. Moreover, related studies show that the main cause of non-Fick diffusion is the formation of water clusters and the water-coating interaction and organic polymers. Therefore, this paper analyzes the causes of non-Fick diffusion under different temperature conditions through immersion experiment.

In this paper, DGEBA (diglycidyl ether of bis phenol A) epoxy coatings were used as a sample to explore the diffusion process of water in 3 wt.% NaCl solution at different temperatures. The diffusion mechanism was explored by immersion experiment and MD (Molecular dynamics) simulation, and the synergistic effect of temperature and water on coating properties was revealed by Fourier infrared (FTIR) and adsorption-desorption cyclic testing.

## 2. Experiment settings

### 2.1 Sample

**Figure 1.**
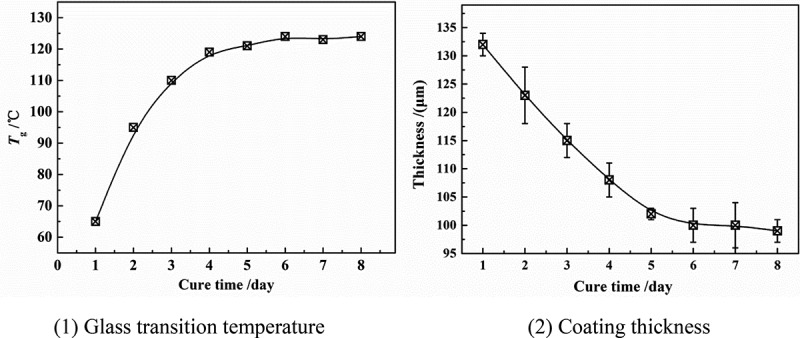
Changes in coating thickness and glass transition temperature over cure time

**Figure 2.**
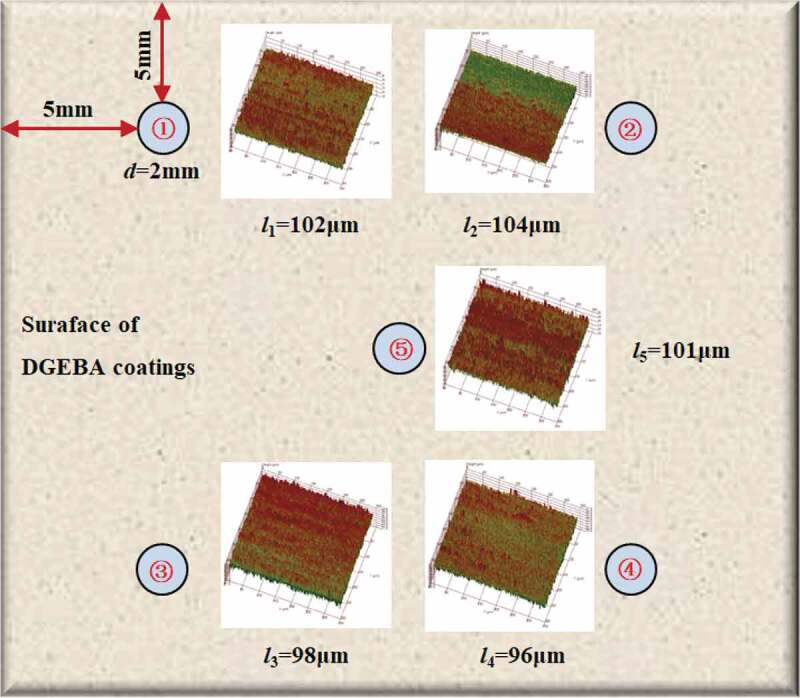
Coating thickness and micro characteristics at five points

The experimental coatings were DGEBA epoxy coatings produced by Anhui Wuhu [2,3,5]. The weight ratio of epoxy resin to curing agent (modified ethylenediamine) was 10:1 and the density was 1.28 g/cm^3^.The coating was applied on the surface of the PE (polyethylene) matrix (25 × 25 mm^2^) [6,7] by hand brush and cured for 7 days at 20 °C. The thickness of the coating sample was determined by QNIX8500 gauge through the five-point sampling method (Figure 2). Figure 1 shows the relationship between coating thickness (*l*) and glass transition temperature (*T*_g_) over curing time. With the prolonging of curing time, the *T*_g_ of the polymer increased gradually, while the polymer thickness decreased gradually, in which *T*_g_ = 123 °C, *l* = 100 ± 5 μm at 7 days. Therefore, after curing at 20 °C for 7 days, the coating sample was considered to be in a stable state, at which the surface of the coating was basically flat, and the thickness difference of the coating at each measurement point area was less than 10 μm, ensuring the homogeneity of the coating, as shown in Figure 2.

### 2.2 Experiment settings

(1) Adsorption

**Figure 3.**
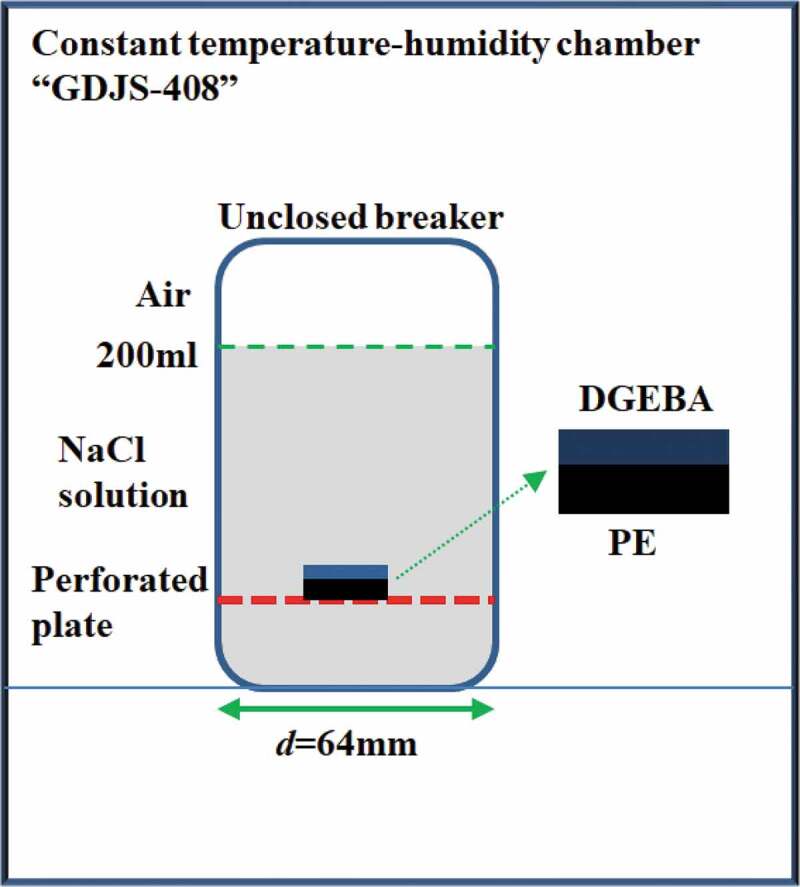
Adsorption test device at different temperatures

The adsorption test was carried out in 3 wt.% NaCl solution at different temperatures (20 °C, 40 °C and 60 °C), the device, as shown in Figure 3. The experiment lasted for 30 days and the total mass of the samples was weighed every 24 h using ML204 analytical balance with an accuracy of 0.1 mg. Three groups of parallel samples were set for each experiment to reduce the experimental error.

The gain of water mass in coatings can be calculated by Eq.(1):

(1) $$\omega_{1}\left(\%\right)=\frac{M_{1}-M_{0}}{M_{0}}\times 100\%$$

where *ω*_1_ is the gain of water mass in coatings in %; *M*_1_ and *M*_0_ are the wet and dry coatings in mg, respectively.

(2) Desorption

After adsorption tests, desorption tests were applied on the aged sample at 60 °C assisted with the vacuum-pumping device in the drying environment. The total mass of the samples was weighed every 1 h until the difference between the two test results was within ±10 mg.

The loss of water mass in the coatings can be calculated by Eq. (2):

(2) $$\omega_{2}\left(\%\right)=\frac{M_{1}-M_{2}}{M_{1}-M_{0}}\times 100\%$$

where *ω*_2_ is the loss of water mass in coatings in %; *M*_1_, *M*_2_ and *M*_0_ are the wet, desorbed and dry coatings in mg, respectively.

(3) MD (molecular dynamic) simulation

The sorbent module in Materials Studio software was used to analyze the energy change process of water diffusion in DGEBA epoxy coatings at different temperatures of 20°C, 40°C and 60°C, respectively. Periodic boundary conditions were set with the task adsorption isotherm selected. Equilibration steps were set as 2,000,000 and the force field was COMPASS. The Ewald and Atom Based methods were used for static force and van der Waals force, respectively.

(4) DSC test

DSC (differential calorimetry scanning) curves of initial samples and experimental samples under different temperature conditions were tested, respectively, to determine the *T*_g_, and then analyze the influence of the synergistic effect of temperature and water on coating properties. The sample weight was 6 ~ 10 mg. The test temperature range was 5–200 °C, and the heating or cooling rate was set at 5 °C/min.

## 3. Results and discussion

### 3.1 Analysis of diffusion process

#### 3.1.1 Continuous model of water diffusion in coatings

(1) Analysis of water diffusion at different temperatures Based on the Fick’s second law, at the initial stage of diffusion process [8,9]:

(3) $$\varphi \left(t\right)=\frac{M_{t}}{M_{\infty }}=\frac{2\sqrt{D}}{l\sqrt{\pi }}\cdot \sqrt{t}$$

where *φ*(t) is the mass fraction of water in coatings at time *t; M*_t_ and *M*_∞_ are the mass of water at time *t* and in saturation, respectively; *D* is the diffusion coefficient, *l* is the coating thickness and *t* is the diffusion time.

**Figure 4.**
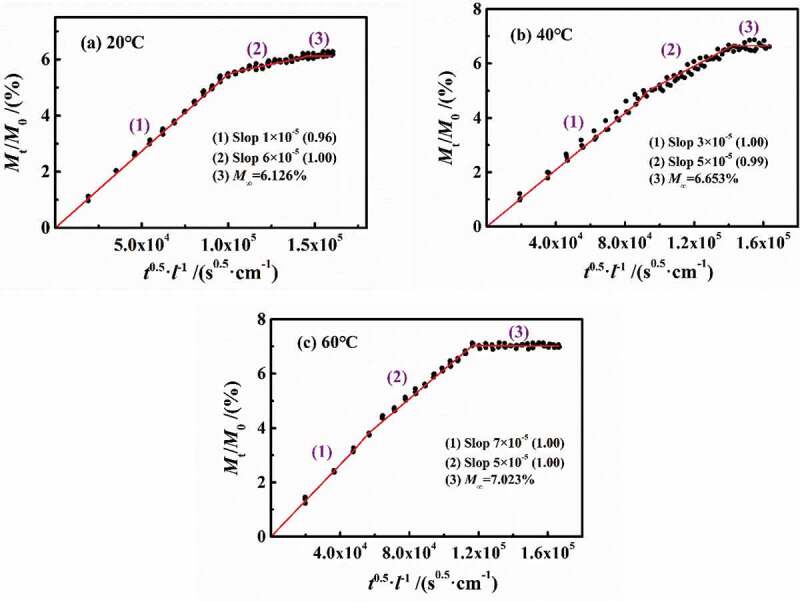
Experimental results of 3 wt.% NaCl solution diffusing in DGEBA coatings on PE base at different temperatures of 20 °C, 40 °C and 60 °C

Figure 4 shows the experimental results of 3 wt.% NaCl solution diffusing in DGEBA coatings on PE base at different temperatures of 20 °C, 40 °C and 60 °C. Under different temperature conditions, the water diffusion behaved obviously as a two-stage process. However, with the increase in temperature, the differentiation degree of two-stage diffusion reduced. In Figure 4, with the increase in temperature, the slopes of stage 1 were 6×10^−5^ (20 °C), 5×10^−5^ (40 °C) and 7×10^−5^ (60°C), while the slopes in stage 2 were 1×10^−5^ (20 °C), 3×10^−5^ (40 °C) and 5×10^−5^ (60°C), showing that the increase in temperature will severely affect the diffusion rate. In combination with the diffusion coefficients at different temperatures in Figure 6, it can be seen that at the experimental temperatures of 20–60 °C in this paper, the change rate of the diffusion coefficient in stage 1 (*D*_1_) was 2.13%, while that of stage 2 (*D*_2_) was 10.40%. Moreover, the *D*_1_ was larger and greater than *D*_2_. Therefore, the effect of temperature on the water diffusion in stage 2 was greater than stage 1. At the same time, with the increase in temperature, the saturated water content increased gradually (6.126%→6.653%→7.023%).

Figure 5

**Figure 5.**
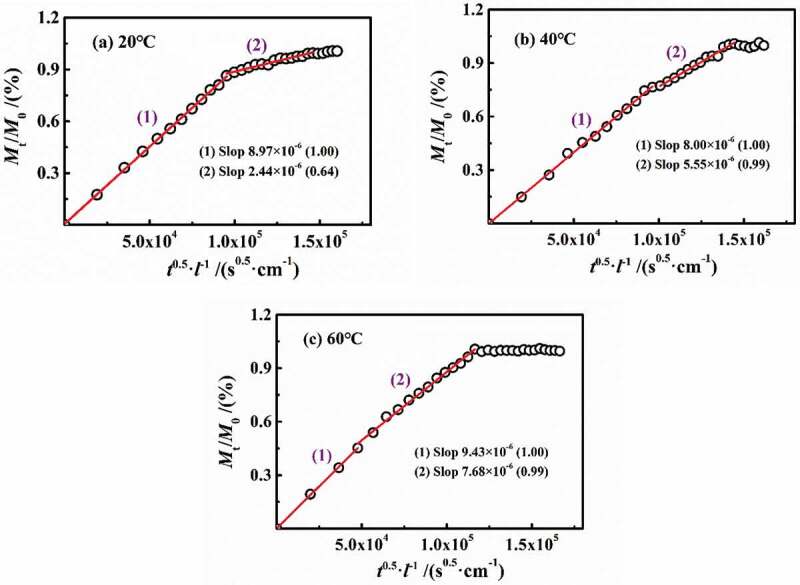
Fitted curves of immersion experiment at different temperatures

**Figure 6.**
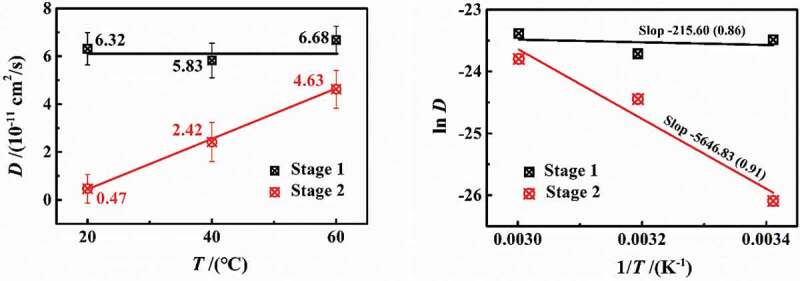
Diffusion coefficient (*D*) and experimental temperature (*T*) at different temperatures

It is well-known that, at the initial diffusion time, water diffused freely in micro-pores, and then the formation of water clusters and water-coating interaction may occur. It was obvious that the above experimental results coincided with this description, but so far, it can be proved in this section. Moreover, it should be noted that the two-stage diffusion may also be caused by the inhomogeneity of the coating structure, that is, the water diffused first at the location with low cross-linking density, and then at the location with higher cross-linking density [10].

Therefore, we assumed that the diffusion process of water in coatings was continuous, that is,stage 2 occurred after stage 1 of diffusion was completed. And the other assumption was that water would not interact with coating molecules during the diffusion process. Based on the above two assumptions, the two-stage process was caused by the inhomogeneous crosslinking density of DGEBA coatings.

(2) Diffusion coefficient at different stages

Considering the coating thickness and edge effect, the *D* was modified by Eq.(4):

(4) $$D=D_{0}\cdot {\left(1+\frac{l}{a}+\frac{l}{b}\right)}^{-2}$$

where *D* is the modified diffusion coefficient in cm^2^/s; *D*_0_ is the calculated diffusion coefficient in cm^2^/s; *l, a* and *b* are the thickness, length and width of samples in mm, respectively.

According to Eqs. (3) and (4), the calculation results are shown in Figures 5 and 6. It can be seen that the *D*_1_ did not change much with the increase in temperature (the average value was 6.27 × 10^−11^ cm^2^/s), while the *D*_2_ increased linearly (0.46→2.38→4.56 × 10^−11^ cm^2^/s), which may be related to the effect of temperature on the coating internal structure.

The increased temperature, enlarging the disorder inside the coatings, will change the free volume in coatings and also improve the elasticity of coating molecules. The internal features, including physical and chemical structure, of the experimental samples under the same curing condition had no difference, that is, once the coating curing had been completed, the internal maximum size of the free volume was also certain, which would not been changed by temperature [11]. Therefore, for the zone with higher crosslinking density, the free volume was small. With the increase in temperature, the improved activity of the coating molecular chain led to a lower resistance for water diffusion, behaving that the *D*_2_ increased linearly.

The relationship of temperature and diffusion coefficient can be described by Arrhenius’ equation [12,13]:

(5) $$D=D_{0}\exp\left(\frac{-E_{a}}{RT}\right)$$

where *D* is the diffusion coefficient in cm^2^/s; *D*_0_ is the constant; *E*_a_ is the activation energy in kJ/mol; *R* is the gas constant in 8.314 J/(mol·K); and *T* is the absolute temperature in K.

**Figure 7.**
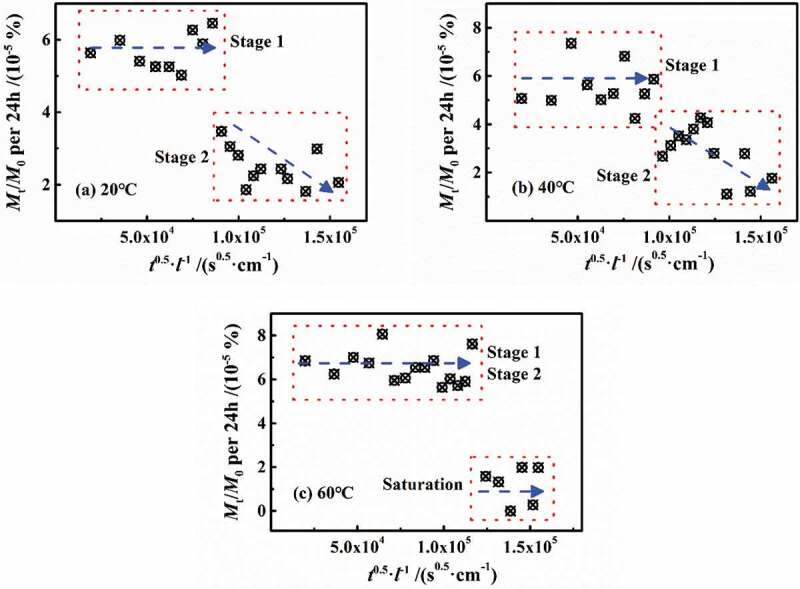
Changes in *M*_t_/*M*_0_ in per 24 h vs. *t*^0.5^/*l* at different temperatures

Taking the logarithm from both sides of Eq. (5), Figure 6 shows the relationship of natural log(*D*)~1/*T* at different temperatures. Linear fitting was performed for two-stage diffusion, respectively. With the gas constant *R* = 8.314 J/(mol·K), the activation energy of the two stages was *E*_a1_ = 1.793 kJ/mol and *E*_a2_ = 46.948 kJ/mol, respectively. The calculated results were consistent with the range of activation energies (10^1^–10^2^ kJ/mol) reported in relevant literatures [7,14,15]. The slope of ln(*D*)~*E*_a_ curve of stage 2 was more larger than stage 1, indicating that stage 1 of diffusion in the lower-density zone was little affected by temperature, which meets with the results of diffusion coefficient.

n most published papers, the defined early stage of diffusion, such as *M*_t_/*M*_∞_<0.6, can be considered as Fick process. Figure 7 shows the variation of *M*_t_/*M*_0_ in per 24 h vs. *t*^0.5^/*l* at different temperatures with a slope of 2(*D*_t_/*π*)^0.5^, in which *D*_t_ was named as the diffusion coefficient per 24 h. At 20 °C and 40 °C, the *D*_t_ in stage 1 can be considered as unchanged, while in stage 2, *D*_t_ gradually decreased prolonging with the water diffusion. At 60 °C, *D*_t_ in both of the two stages was constant and equal, and then *D*_t_≈0 in saturation.

The statistical test of the diffusion coefficient per 24 h (*D*_t_) in the second stage was carried out at different temperatures. The results showed that *F* (72.51) > *F* crit (3.49) and *P* (6.84 × 10^−10^) < 0.01, indicating that the diffusion coefficient values for stage 2 were significantly different than each other at the various temperatures. Theoretically, the effect of temperature on the coating of molecular chains at high cross-linking density would have no obvious difference, indicating that the *D*_t_ of every 24 h in stage 2 should be the same, which was far away from the experimental results. Therefore, it was inferred that the aggregation and relaxation processes may occur during stage 2, which was contrary to the assumptions of the above analysis. The reasons for this phenomenon will therefore be discussed below.

(3) Analysis of the coating swelling coefficient per unit time (24 h)

**Figure 8.**
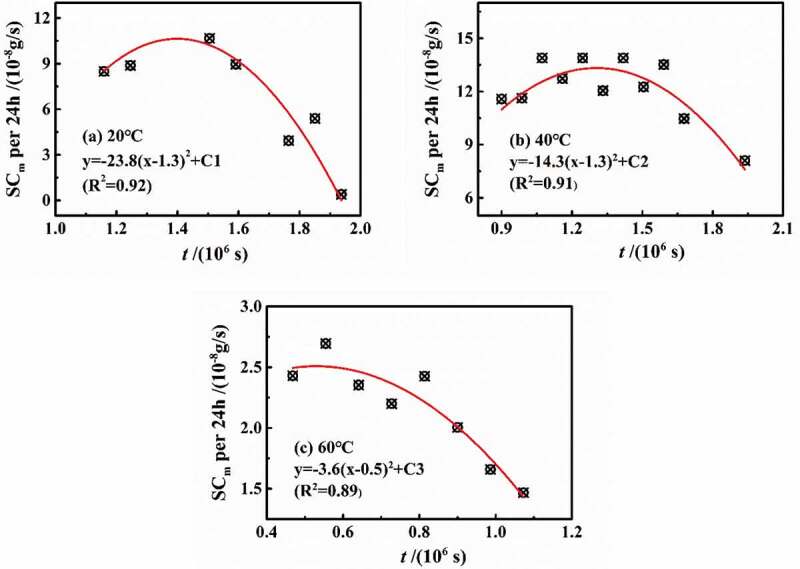
Changes in the swelling coefficient per 24 h named as SC_m-t_ at different temperatures

According to the above analysis, the influence of temperature on the coating structure was mainly aimed at the location of the higher crosslinking density. However, the mass fraction of water increased over diffusion process, which would cause the swelling phenomena in the coating volume, especially for the high-density zone. Based on the Fick diffusion, Westing et al. [16] proposed the swelling coefficient (SC_m_) to represent the gain of water mass caused by the coating swelling, and established a linear relationship model between swelling coefficient and diffusion time. In this paper, the swelling coefficient revealed the average value during stage 2. Furthermore, the change in the curves of swelling coefficient in per 24 h (SC_m-t_) may present different rules, as shown in Figure 8, in which the data were in the range of stage 2 at different temperatures. The variation of SC_m-t_ was similar to that of *D*_t_, which varied with time rather than a theoretical unchanged value. For another aspect, swelling coefficient was related to the change in coating thickness caused by physical and chemical processes, which would be further focused on the swelling caused by water clusters and relaxation process.

Figure 8 shows the fitted equations of SC_m-t_ at different temperatures, all of which were in the form of quadratic functions. To our best knowledge, the rising temperature may cause the state of coatings transforming glassy state to rubbery state. According to H.L. Frisch’s classification of diffusion processes [17], it can be inferred that water was more likely to undergo relaxation reactions at 20 °C than that at 60 °C. When the water interacted with the coating molecules, the coating chain was opened to reduce cross-linking density, indicating that SC_m-t_ increased. On the other hand, when multiple water molecules gathered together to form a water cluster, the volume occupied by *n* individual water molecule was less than the volume of the water cluster formed by *n* water molecules, showing that SC_m-t_ decreased [11]. Therefore, it can be seen from the fitted curves in Figure 5 that under different temperature conditions, in stage 2, the water-coating interaction occurred first, followed by the formation of water clusters.

Based on the fitted equations, the influence of temperature on the water diffusion was mainly reflected in two aspects: quadratic-term coefficient and symmetry axis parameter.

(1) The quadratic coefficient reflected the relaxation process of water and coating molecules at different temperatures and also showed the effect of temperature on the high-density zone. The higher the temperature was, the lower the density of the coating molecular chain was. For example, at 60 °C, *D*_2_ tended to be the same as that of stage 1, meaning that the change rate of SC_m-t_ was lower, which can be approximately assumed that the change rate of SC_m-t_ decreased by about 2% for every 1 °C increase in temperature.

(2) The symmetry-axis parameter represented the transition time during the water-coating interaction and the formation of water cluster. At 20 °C and 40 °C, the transition time was all 1.3 × 10^6^ s, while the transition time was 5.30 × 10^5^ s at 60 °C. Therefore, in stage 2, the relaxation process lasted for 1.84 × 10^5^ s, 4.04 × 10^5^ s and 3.0 × 10^4^ s, respectively, at different temperatures. However, it should be noted that the polymerization time of water molecules was all about 5.5 × 10^5^ s.

Generally speaking, when the temperature was higher than the glass transition temperature of coatings, the diffusion process of water satisfied Fick diffusion. Therefore, it was inferred that the water-coating interaction time must decrease as the temperature increased. However, according to the experimental results at 20 °C and 40 °C, the interaction time at 40 °C was longer than 20 °C. Therefore, it was necessary to comprehensively consider the change in high-density zone and relaxation velocity of coating at different temperatures to explore this phenomenon.

At 20 °C, the resistance of water diffusing at the higher cross-linking density zone was larger, and the number of interaction point with coating molecules was less. As the experimental temperature rose to 40 °C, the higher cross-linking density gradually decreased, and more interaction points were exposed. Under this circumstance, the water-coating interaction positions increased significantly, showing that the interaction time was longer than that at 20 °C. When the temperature reached 60 °C, the density distribution in the whole coating system was more uniform, causing an accelerated diffusion and interaction processes. Once there was enough water, it entered the formation stage of water clusters. The above analysis was based on the assumption of the occurrence in order of relaxation and water clusters, which resulted in the main error of the fitted curves. Maybe the different process of interaction or water clusters was simultaneous, but dominated by one process.

Under the condition of different temperatures, the mass fraction of water in coatings was 5.5% (20 °C), 4.9% (40 °C) and 3.9% (60 °C) at the end of stage 1. Under different temperature conditions, the higher the temperature was, the earlier the diffusion process into stage 2 was. However, the formation of water clusters occurred at the same time. Relevant studies [18,19] showed that the structure of water clusters may be different at different temperatures, and the entropy of water clusters increased with the increase in temperature, indicating that the higher the temperature was, the more unstable the water clusters will be. Therefore, the higher the temperature, the more stable water cluster structure will be formed by more water molecules.

#### 3.1.2 Molecular dynamics simulation of water diffusion

(1) Error analysis of MD numerical calculation results and experimental values

In order to explore the energy change caused by water diffusion in coatings, the amorphous structure of the DGEBA coatings was established through molecular dynamics simulation software, and then, the changes in the related energy parameters were analyzed.

**Figure 9.**
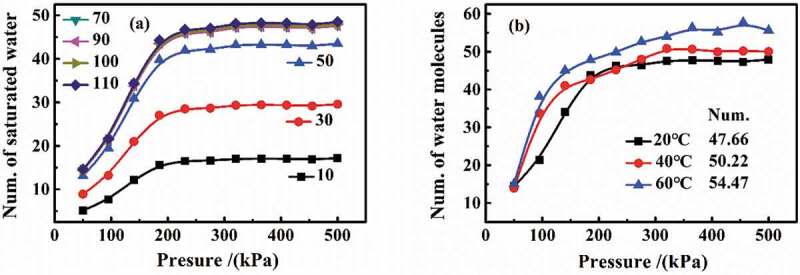
Determination of number of DGEBA molecules in per cell and the number of water molecules in saturation in per cell: (a) the change in the number of saturated water molecules in different DGEBA-molecule number per cell; (b) the number of saturated water molecules per cell containing 100 DGEBA molecules

In the simulation, the different number of DGEBA molecules was set in per amorphous cell, and then structure optimization and energy optimization were carried out. Under the conditions of isothermal adsorption and pressure of 50–500 kPa, the effect of the number of DGEBA molecules per amorphous cell on water diffusion was analyzed. As can be seen from Figure 9 that with the increase in the set pressure, the numbers of water molecules first increased and then tended to be stable, illustrating a saturation state. In detail, in the range of 10–70 DGEBA molecules per cell, more water diffused into coatings, and then, the content of water basically remained unchanged in the range of 70–110. Therefore, 100 DGEBA molecules per cell were set to research the water diffusion. Moreover, the average number of water molecules at saturation per cell under different temperature conditions were also simulated in Figure 9. With the increase in pressure, the saturated numbers of water molecules first increased and then kept flat. The average number of water molecules at saturation was 47.66 in 20 °C, 50.22 in 40 °C and 54.47 in 60 °C.

In Section 3.1.1, the saturated water content was 6.126% at 20 °C, 6.653% at 40 °C and 7.023% at 60 °C, respectively. The error of experimental and simulated results should be calculated to verify the accuracy of the model.

For the isothermal diffusion process, the saturated water content can be calculated through saturated numbers of water molecules using Eq. (6):

(6) $$M_{t}/M_{0}=n/N÷m_{0}\times 18$$

where *n* is the average number of water molecules at saturation per cell, *N* is the Avogadro’s number (6.02 × 10^23^), *m*_0_ is the mass per cell (*m*_0_ = 2.5 × 10^−20^ g) and 18 is the molar mass of water in g/mol.

Table1 shows the error of simulated results compared with experimental results. The maximum error at different temperatures was less than 10%, indicating that MD simulation can well analyze the diffusion process of water in DGEBA coatings.

**Table 1.** Error analysis of numerical simulation

Temperature/°C	Saturated water content/%		
	Simulation	Experiment	Error
20	5.70	6.126	6.95
40	6.01	6.653	9.66
60	6.52	7.023	7.16

(2) Analysis of variable energy parameters during water diffusion

**Figure 10.**
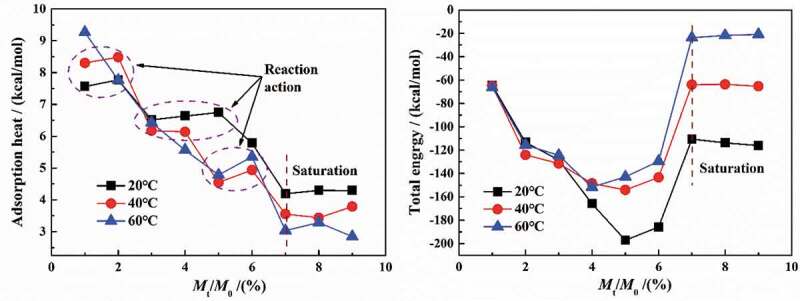
Changes in the related parameters at different temperatures

Figure 10 shows the changes in the average adsorption heat and total energy with the increase in water molecule content at different temperatures. This can be seen from the curve of average adsorption heat, with the increase in water content in coatings, the average adsorption heat was generally reduced, suggesting that the effect of coatings on water diffusion decreased. Moreover, the higher the temperature was, the faster the extent of decrease was.

For the liquid adsorption process, the changes in energy can be described by the Freundlich equation [20] as follows:

(7) $$Qq^{\frac{1}{n}}$$

where *Q* is the adsorption heat in kcal/mol, *q* is the adsorption quantity in mol and *n* is the constant, reflecting the adsorption strength.

From the fitted equations, the coefficient of the fitted equations increases with the increase in temperature (8.8→9.8→10.7). Therefore, the higher the temperature was, the weaker the water-coating interaction was, which met with the results of swelling coefficient in per 24 h (SC_m-t_).

According to the calculation results of MD simulation, at 20 °C and atmospheric pressure, the adsorption heat of single molecule was −0.543 kcal/mol (water-coating) and 5.194 kcal/mol (water-water), respectively, showing that the water-coating interaction was an exothermic process, while the forming water clusters was an endothermic process.

Therefore, at 40 °C and 60 °C, the increase in adsorption heat between 5% and 6% (*M*_t_/*M*_∞_≈0.7) was the result of the water-coating interaction, and the subsequent decrease in adsorption heat was the synergistic result of the formation of water clusters and the reduction of diffusion rate. However, for the adsorption heat curve at 20 °C, the adsorption heat continued to decrease in 5%–7%, suggesting that the interaction process may have occurred at this time. The same experimental phenomena occurred in the water content range of 1%–2% at 20 °C and 40 °C. The following corollary can be drawn from the above analysis. Generally, the relaxation process did not occur after the water molecules had diffused to a certain extent, but may occur locally.

For another aspect, when the water content was more than 7%, showing a saturated state, the adsorption heat basically remained unchanged with an increasing trend at 20–60 °C, which was caused by the differently influenced degree of DGEBA chains with higher cross-linking density at different temperatures.

The diffusion process of water in coatings began with the free diffusion process in the pores, meaning a filling process of the free volume. Due to the narrow diameter of the pore diffusion channel and the closer distance between the adjacent pore walls, the superposition of the pore wall potential field made the diffusion process of water molecules highly dependent on the pore size and the force at the adsorption interface. The physical diffusion of water molecules in DGEBA system was mainly determined by the electrostatic force and van der Waals force. Among them, electrostatic force mainly referred to the interaction force between hydrogen bonds, while van der Waals force referred to all inter-molecular forces except the electrostatic action of ions and hydrogen bonds. With the increase in water content, both the van der Waals force and the electrostatic force increased exponentially, but the total energy first increased and then decreased in a parabolic pattern.

During the diffusion of water, the van der Waals force acted as repulsion, while the electrostatic force acted as attractive force. With the increase in water molecules in 0–5%, the increase in electrostatic force was greater than that of van der Waals force, which was manifested as the increase in total energy. However, two aspects should be paid attention to in different diffusion processes:

(1) The formation of water clusters was an endothermic reaction, leading to a reducing heat absorption, such as the case of 5%~7% (water content) at 40 °C and 60 °C. The relaxation process was an exothermic reaction, resulting in an increasing heat absorption, such as the case of 3%~5% (water content) at 20 °C.

(2) Once the water content in coatings reached saturation, the total energy no longer changed. However, the ‘saturated’ total energy was variable at different temperatures, which was due to the differently influenced degree of DGEBA chains with higher cross-linking density at different temperatures. For example, the coating was more rubbery at 60°C than at 20°C, meaning that a dynamic balance trended to be reached, in which the total energy was close to zero.

Fitted equations of adsorption heat at different temperatures:

$$\begin{array}{c} y=8.8\cdot x^{-0.292}\left(R^{2}=0.71\right)\left(20^{\circ }C\right)\\ y=9.8\cdot x^{-0.426}\left(R^{2}=0.78\right)\left(40^{\circ }C\right)\\ y=10.7\cdot x^{-0.542}\left(R^{2}=0.87\right)\left(60^{\circ }C\right) \end{array}$$

#### 3.1.3 Simultaneous model of water diffusion in coatings

The continuous model of water diffusion in coatings was based on the assumption that the non-Fick diffusion occurred in stage 2. However, the MD simulated results showed an amazing phenomenon that the non-Fick process occurred at stage 1, especially at 20 °C and 40 °C.

**Figure 11.**
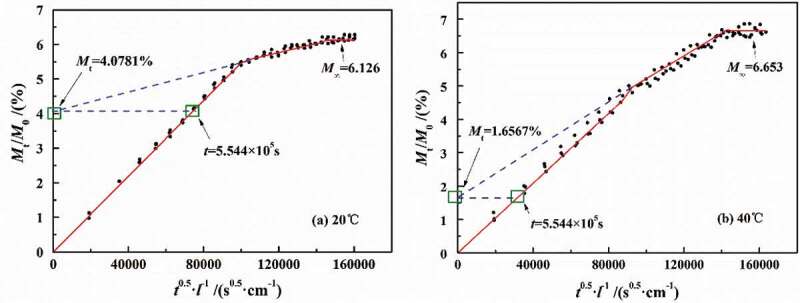
Water contents and diffusion time of initial diffusion stage at 20 °C and 40 °C

Figure 11 shows the fitted results of the simultaneous model. It can be seen that the diffusion process meeting with Fick process was *M*_t_/*M*_0_ = 0 ~ 4.0748% within diffusion time *t* ≈ 0–5.544 × 10^5^ s at 20 °C and *M*_t_/*M*_0_ = 0–1.6567% within diffusion time *t* ≈ 1.224 × 10^5^ s at 40 °C. The above results seemed to be consistent with the stage of Fick diffusion (*M*_t_/*M*_∞_<0.6) proposed by relevant scholars. However, the difference of *M*_t_/*M*_∞_ satisfying Fick diffusion obtained at 20 °C and 40 °C in this paper was 2.67 times. At the same time, the analysis results showed that the lower the temperature was, the longer the Fick stage was, which was contrary to the related theory of coating property–temperature relationship.

Up to now, many scholars considered the further diffusion of water molecules caused by the relaxation reaction and water clusters. Under these circumstances, the swelling coefficient (*S*) was only 1%–3% of the diffusion coefficient. However, the *S* value was far greater than 3%. As mentioned above, the occurrence of two-stage diffusion process was not only related to the non-Fick process but also to the diffusion process of water in different density regions. From the results of water gain-loss experiment, with the increase in temperature, the *D*_1_ basically remained unchanged, while the *D*_2_ gradually increased. Therefore, the occurrence of the two-stage diffusion process was mainly caused by the effect of temperature on the physical structure of the DGEBA coatings.

### 3.2 Analysis of synergistic effect of temperature and water

#### 3.2.1 Analysis of water desorption

**Figure 12.**
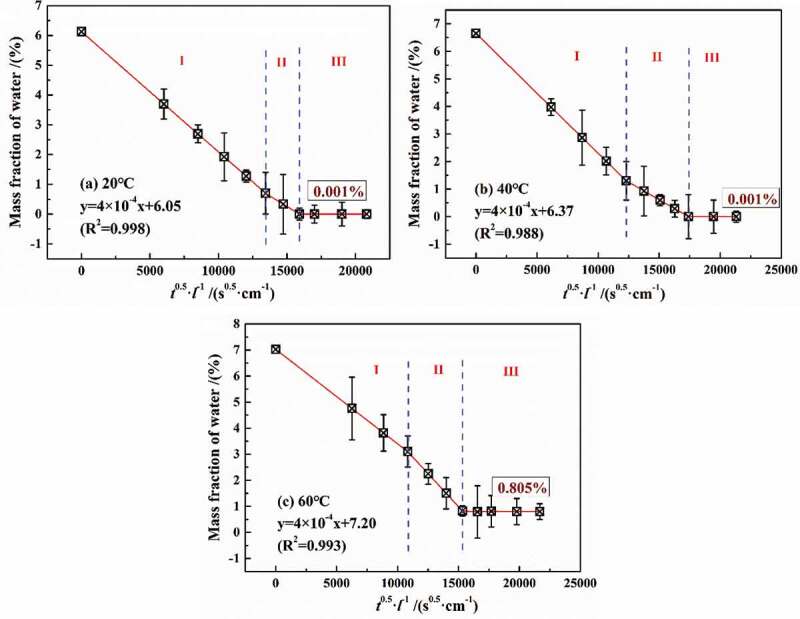
Experimental results of water molecule desorption from polymers on PE base

According to the Eq.(3), Figure 12 shows the desorption results of water in DGEBA coatings in a drying environment of 60 °C with an auxiliary vacuum device. Similar to the adsorption results in Figure 4, it can be divided into three stages, in which the rate of water loss (desorption coefficient) in stage 2 was slightly lower than that in stage 1. This was because that different temperature made a difference in high-density zone in water diffusion. However, the desorption test was carried out at 60 °C, reducing the difference in desorption coefficient, that is, the difference in desorption coefficient between the two stages can be ignored. Therefore, the total fitted equations of stage 1 and stage 2 were shown in a good linear relationship with the variance greater than 0.98.

Based on the fitted line slope, the desorption coefficient was calculated to 1.257 × 10^−5^ cm^2^/s, which was far greater than the diffusion coefficient at different temperatures, indicating that water diffusion in coatings was needed to overcome the blocking effect of DGEBA chains. Therefore, water diffusion can be considered as micro-pore adsorption, which can be described by the D-R equation to calculate the volume of the micro-porous system.

The D–R equation was proposed based on the Polanyi adsorption theory, which was only applicable to physical adsorption due to its thermodynamic theory [21]. Therefore, the total volume of micro-pores in coatings can be calculated by the following Eq. (8):

(8) $$\mathrm{lg}W=\mathrm{lg}W_{0}-B\mathrm{lg}{\left(P_{0}/P\right)}^{2}$$

where *W* is the micro-porous volume at relative pressure *P*/*P*_0_ in cm^3^; *W*_0_ is the micro-porous volume in cm^3^; *P*_0_ is the saturated vapor pressure of water, equal to 2.34 kPa (20 °C), 7.38 kPa (40 °C) and 19.93 kPa (60 °C); *P* is the calculated pressure in kPa; *B* is the constant related to the physical structure and temperature.

**Figure 13.**
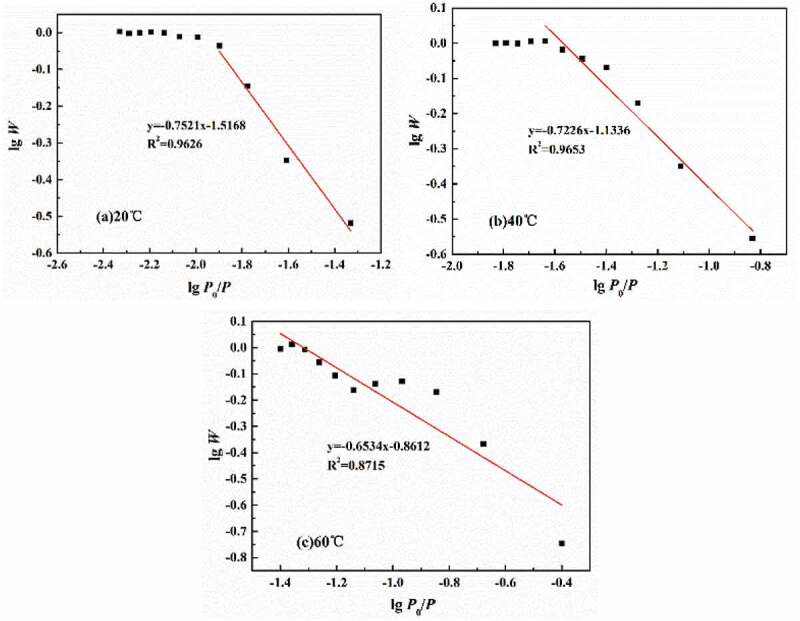
lg*W*-lg(*P*_0_/*P*) curves at different curves

It is assumed that once the water diffusion in coatings reached saturation, all the free volumes of micro-pores were occupied. Therefore, the volume of micro-pores can be expressed as the ratio of the number of water under certain pressures to the number of saturated water. According to Eq. (8), lg*W*-lg(*P*_0_/*P*) curves at different temperatures can be obtained, as shown in Figure 13. Therefore, according to the slope of the fitted lines, the total volume of micro-pores in polymers at different temperatures was 21.9% (20 °C), 32.2% (40 °C) and 42.3% (60 °C), respectively, meaning that for every 1 °C increase in experimental temperature, the total volume of micro-pores was increased by 0.51%.

However, from the results of water desorption, at 20 °C and 40 °C, water can completely desorb from the coatings with less than 0.001% (mass fraction), while the mass fraction of the remaining water was 0.805% at 60 °C, showing that temperature may have an effect on the physical structure of coatings. Relevant studies have shown that, at a higher temperature, water was likely to cause the fracture of DGEBA chains, and then the water will combine with the broken functional groups. On the other hand, with the increase in temperature, the thermal expansion effect of the coating volume will lead to the irreversible transformation of the free volume in coatings, which was conducive to promoting the diffusion of water. Considering the synergistic effect of temperature and water on coatings, it may cause the precipitation of some low-weight molecular components or unattached substances, showing that the dry weight of the desorbed coating was less than its original weight. Therefore, the following paper focused on the effects of different temperatures and water on the physical/chemical properties of the coatings.

#### 3.2.2 Analysis of different temperature

(1) Analysis of the effect of higher temperature (60 °C) via DSC and FTIR

**Figure 14.**
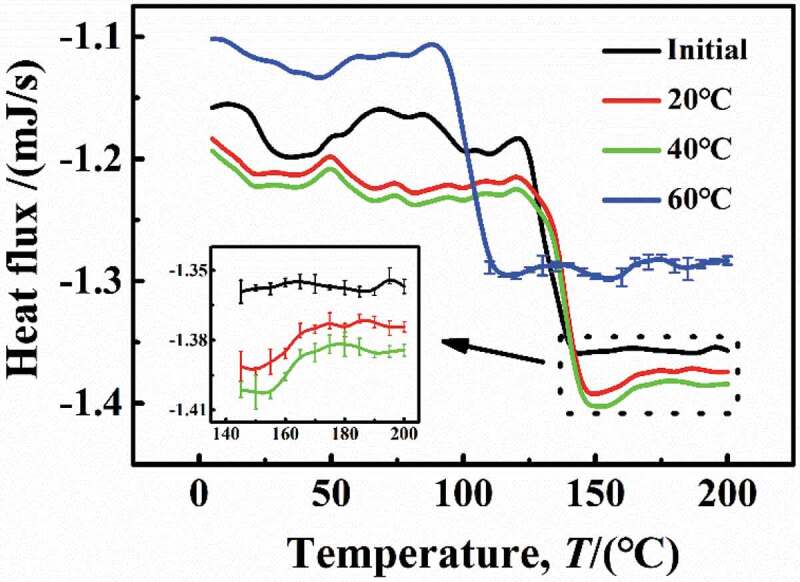
DSC results of the experimental samples at different temperatures

The *T*_g_ reflected the degree of cross-linking of polymer chain [22]. Figure 14 shows the DSC results of the DGEBA coatings at different temperatures, and it can be seen that the *T*_g_ of the initial sample was 123 °C. Under experimental conditions of 20 °C and 40 °C, the DSC curves were in consistent with the initial sample with a higher heat flow density and the *T*_g_ = 123 °C, suggesting that under these conditions, the water molecules in coatings can promote the formation of hydrogen bonds, but there was no cause between the DGEBA chain rupture or cross-linking again. However, at 60 °C, the DSC curve was different from that under low temperature (20 °C and 40 °C), the heat flux required decreased, and the *T*_g_ was significantly reduced to 94 °C, indicating that the destruction of the DGEBA chains may lead to the fracture of chemical bonds, leading to the reduction of the DGEBA cross-linking density.

**Figure 15.**
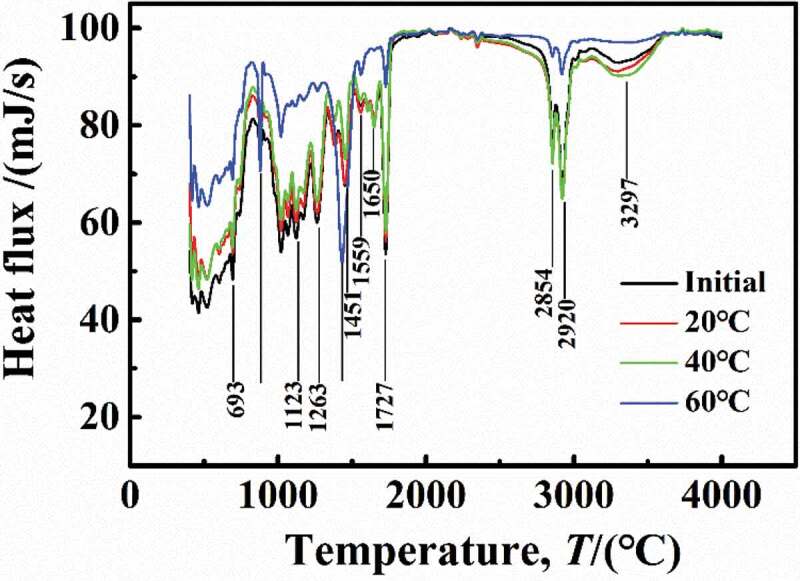
Results of Fourier infrared test of experimental samples at different temperatures

Figure 15 shows the Fourier transform infrared spectroscopy results for samples at different immersion temperatures. At the saturation stage, compared with the initial sample, the adsorption peaks of the samples immersed at 20 °C and 40 °C were located at the same position but increased, which may be caused by the bulge of the number of hydrogen bonds in coatings due to water diffusion. However, at 20 °C and 40 °C, water diffusion did not lead to the fracture of DGEBA chains, which coincided with the results of desorption, that is, at lower temperature, water can completely desorb from the coatings, characterized by a physical diffusion process.

However, at 60 °C, the peaks at several locations were different from the initial sample. The synergistic effect of temperature and water on DGEBA structure was mainly reflected in the following aspects:

(1) There were strong characteristic peaks between 3000 and 3600 cm^−1^ that represented the -OH and -NH functional groups, which indicated the presence of hydrogen bonds in coatings. However, at 60 °C, the new characteristic peaks appeared at 879 cm^−1^ and 1559 cm^−1^, meaning the damage and precipitation of -OH and -NH functional groups [23, 24].

(2) At 60 °C, the characteristic peak disappears at 1650 cm^−1^, indicating ether-group fracture, which made the characteristics of the diphenyl ring (1433 cm^−1^) more obvious.

Therefore, the synergistic effect of higher temperature and water on DGEBA structure was aimed at improving the water-coating interaction to cut off the DGEBA chains and then accelerating the degradation and aging of the coatings, which was same as the experimental results of desorption of water at 60 °C.

(2) Analysis of effect of lower temperature (20 °C) via adsorption-desorption cyclic experiments

As can be seen from the foregoing, water diffusion did not lead to the fracture of functional groups in coatings at 20 °C, which can be inferred that water can always achieve complete desorption in adsorption-desorption experiments. This reflected the fact that the amount of free volume in coatings was constant and did not change due to the adsorption-desorption of water molecules.

**Figure 16.**
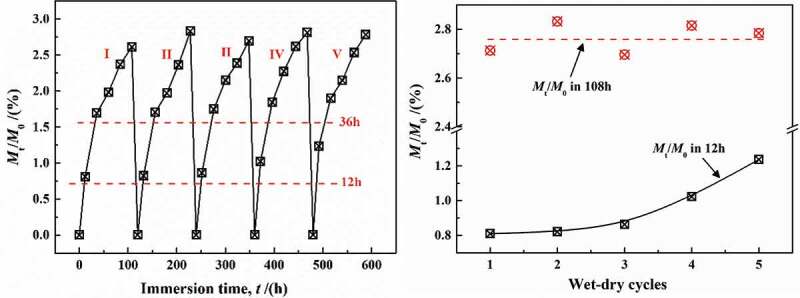
The relationship between water content in coatings with the number of adsorption-desorption cycles at 20 °C

Figure 16 shows the change in the gain or loss of water mass in coatings with the number of adsorption-desorption cycles at 20 °C, mainly showing three characteristics:

(1) During the adsorption-desorption cycles, water can be completely removed from the coatings, and the dry weight of the coatings after every desorption experiment was basically the same as the initial weight, which met with the results in Section 3.2.1.

(2) With the increase in cycling times, the diffusion coefficient in 0–12 h increased obviously, which was reported in previous papers [25–28]. This phenomenon was mainly related to the internal stress caused by the diffused water in coatings [29], i.e., the formation of water clusters expanded the local-free volume, leading to the irreversible damage to physical structure of the DGEBA chains. However, other scholars have put forward that, in the initial stage (0–12 h), the increase in diffusion coefficient may be due to the transition of the connection between the DGEBA chain sites. The diffusion of water increased liquidity of DGEBA chains, making more connecting sites exposed. Therefore, the breaking and recombination of DGEBA chains promoted free volume aggregation to form a greater free volume, which accelerated the rate of diffusion of water molecules in the initial stage [30–33].

(3) The presence of more adsorption sites will increase the crosslinking density of the coatings, which will undoubtedly reduce the diffusion capacity of water [34]. However, there was no similar phenomenon in this paper. Under different cycle times, the diffusion coefficient of water in the coatings (0 ~ 108 h) basically remained unchanged, with an average value of *M*_t_/*M*_∞_ = 2.748%. Therefore, at lower temperature (20 °C), the increase in diffusion coefficient in initial time (0 –12 h) was caused by irreversible [35] change due to adsorption-desorption cycles, leading to fatigue in the physical structure of coatings [23].

## 4. Conclusion

In this paper, DGEBA epoxy coating was used as the sample to explore the diffusion process and the synergistic effect of temperature and water molecules on the DGEBA properties of 3 wt.% NaCl solution at different temperatures. Three conclusions were drawn as follows.

(1) With the increase in experimental temperature, the saturated water content in coatings gradually increased to 6.126% (20 °C), 6.653% (40 °C) and 7.023% (60 °C), respectively. The diffusion process of water included two stages, in which the diffusion coefficient was constant (*D*_1_ = 6.013 × 10^−11^ cm^2^/s) in stage 1 and increased in stage 2 (*D*_2_ = 0.459→2.383→4.558 × 10^−11^ cm^2^/s), indicating that the influence of temperature on the diffusion process was mainly applied on the DGEBA chains with higher crosslinking density.

(2) According to the change law of swelling coefficient per 24 h (SC_m-t_), the conditions for water-coating interaction and formation of water clusters in this paper were proposed, showing that the duration of the interaction was related to temperature, while the formation of water clusters was only related to the number of water in coatings.

(3) Through the adsorption–desorption cycles and Fourier infrared tests, the synergistic effect of water and temperature on DGEBA properties was analyzed. The results showed that at lower temperature (20 °C), water would only change the physical structure of the coatings, while the water broke the DGEBA chains at higher temperature (60 °C).
